# Tislelizumab-induced fulminant type 1 diabetes mellitus (FT1D): A case report and literature review

**DOI:** 10.1097/MD.0000000000046310

**Published:** 2025-11-21

**Authors:** Xipeng Zhou, Peng Jiang, Mingxiang Chen, Jiashun Chen

**Affiliations:** a Department of Oncology, Yizheng People’s Hospital, Yizheng, Yangzhou, Jiangsu, China.

**Keywords:** diabetes ketoacidosis, fulminant type 1 diabetes mellitus, immunotherapy, PD-1, tislelizumab

## Abstract

**Rationale:**

Immune checkpoint inhibitors, such as tislelizumab, are widely used in oncology but can cause serious immune-related adverse events. Endocrine disorders are common, with fulminant type 1 diabetes mellitus (FT1D) being a rare but life-threatening complication. This case highlights the rapid onset of FT1D following tislelizumab therapy.

**Patient concerns:**

An elderly male with lung squamous cell carcinoma, with no prior history of diabetes, was admitted to the hospital complaining of systemic malaise, nausea, and vomiting.

**Diagnosis:**

Laboratory findings confirmed diabetic ketoacidosis (DKA), hyperkalemia, and hyponatremia. Further assessment revealed severely depleted C-peptide levels (both fasting and postprandial <0.05 ng/mL) and an HbA1c of 7.7%, with negative islet autoantibodies. A diagnosis of tislelizumab-induced fulminant type 1 diabetes mellitus was established.

**Interventions:**

The patient was managed for DKA with fluid resuscitation, continuous intravenous insulin infusion, and electrolyte correction.

**Outcomes:**

The patient’s metabolic abnormalities were corrected, and his blood glucose levels stabilized under the subcutaneous insulin regimen.

**Lessons:**

Tislelizumab can induce FT1D, which presents acutely and can be fatal. This case underscores the critical need for vigilant and regular blood glucose monitoring in patients receiving immune checkpoint inhibitors. Enhanced patient education on the symptoms of hyperglycemia is essential to enable early detection and prompt intervention, thereby preventing severe complications such asDKA.

## 1. Introduction

**Figure 1.**
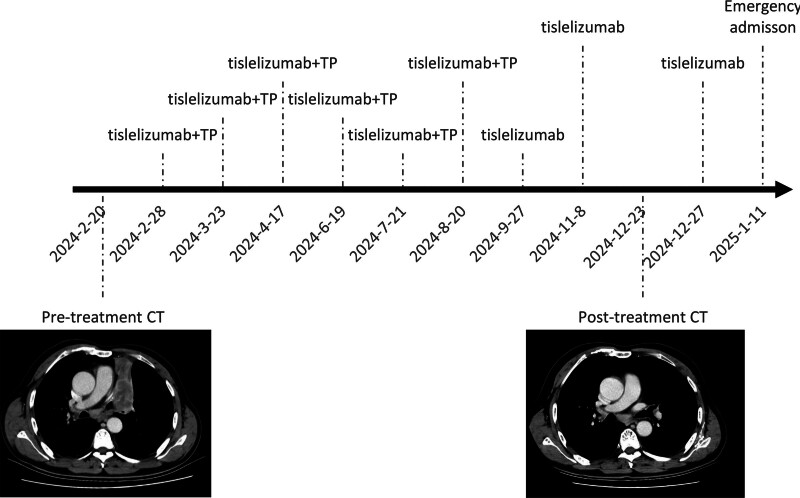
The entire antitumor treatment timeline of the patient, along with the chest CT mediastinal window manifestations at the time of initial diagnosis and after the completion of standard 6-cycle hybrid chemo-immunotherapy, followed by single-agent immunotherapy. CT = computed tomography.

**Figure 2.**
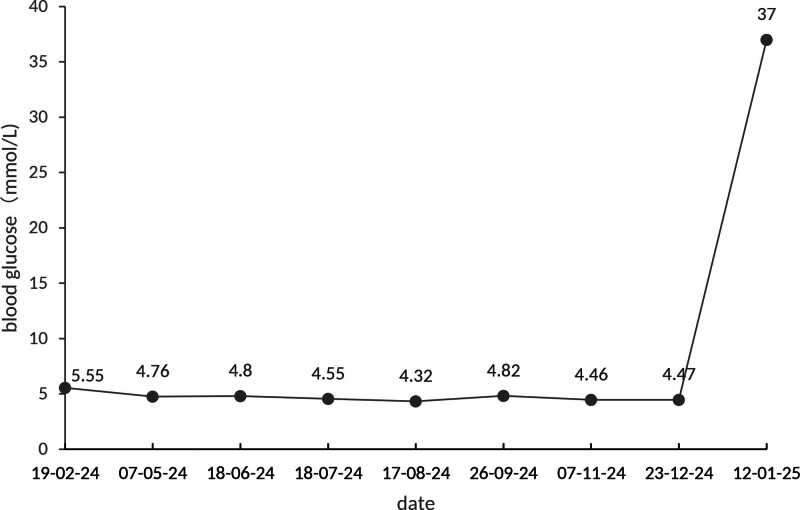
The daily fasting blood glucose fluctuations of the patient and the capillary blood glucose value measured upon emergency admission on the day of onset.

The patient is an elderly male who initially presented with cough and sputum production. A chest computed tomography scan in February 2024 showed a mass in the left upper lobe, along with enlarged hilar and mediastinal lymph nodes. On February 20, a lung puncture biopsy revealed a pathological diagnosis of squamous cell carcinoma. Immunohistochemistry findings were as follows: tumor cells were P40 (+), P63 (+), CK5/6 (+), TTF-1 (–), NapsinA (–), Syn (–), and Ki-67 (50%+). The patient received first-line treatment at a private hospital with 3 cycles of tislelizumab combined with nab-paclitaxel and nedaplatin. During this period, he developed severe grade 3 bone marrow suppression. Later, at our hospital, the treatment regimen was changed to tislelizumab combined with docetaxel and nedaplatin for 3 cycles. Since September 27, 2024, the patient has been receiving maintenance immunotherapy with tislelizumab as a single agent (Fig. 1). On January 11, 2025, the patient was admitted with systemic malaise, nausea, and vomiting. He had no prior history of diabetes (Fig. 2). Emergency laboratory results confirmed diabetic ketoacidosis, hyperkalemia, and hyponatremia (Table 1). In follow-up examinations after hospitalization, his fasting and 2-hour postprandial C-peptide levels were both below 0.05 ng/mL, hemoglobin A1c (HbA1c) 7.7%, and islet autoantibodies tests returned negative results. Treatment included fluid resuscitation, insulin infusion (0.1 international unit [IU]/kg/h), electrolyte correction, and transition to a basal-bolus insulin regimen (aspart 8 IU premeal; glargine 12 IU nightly). Finally, his blood glucose stabilized under insulin therapy.

**Table 1:** Initial diagnostic findings at the time of emergency admission.

Test parameter	Value	Reference range
Oxygen Inhalation (%)	33.0	
Body Temperature (°C)	36.8	36~37
Oxygen Saturation (%)	99.0	95~99
pH (37°C)	6.98	7.35~7.45
pCO₂ (mm Hg)	14.9	35~48
pO₂ (mm Hg)	145	83~108
HCO₃⁻ (mmol/L)	3.5	21~28
Standard HCO₃⁻ (mmol/L)	7.1	22.5~26.9
Hematocrit (%)	42.5	37~49
Oxyhemoglobin (%)	98.2	93~98
Deoxygenated hemoglobin (%)	1.0	2~7
Total hemoglobin (g/dL)	13.9	13.5~17.5
Potassium (mmol/L)	6.8	3.5~4.5
Sodium (mmol/L)	116	136~146
Ionized calcium (mmol/L)	1.21	1.15~1.29
Glucose (mmol/L)	37	3.9~5.8
Lactate (mmol/L)	2.1	0.5~1.6
Base excess (mmol/L)	−26.6	−3~3
Anion g(mmol/L)	21.4	8~12
Chloride (mmol/L)	92	96~108
Urine ketones	3+	Negative
Urine glucose	4+	Negative

## 2. Discussion

In 2015, Martin-Liberal et al^[1]^ first reported a case of autoimmune diabetes in a patient with cutaneous melanoma following pembrolizumab therapy. With the widespread clinical application of immune checkpoint inhibitors (ICIs) in oncology, the incidence of ICI-associated diabetes mellitus (ICI-DM) has gradually increased in recent years, with a reported prevalence of 0.2% to 1.9%.^[2–4]^ The onset of ICI-DM varies widely, ranging from weeks to over a year after initiating treatment, with a median time to onset of 20 weeks.^[3,5]^ The underlying mechanisms of ICI-DM remain poorly understood. Animal studies suggest that blocking programmed cell death protein 1 and its ligand disrupts immune regulatory processes, leading to autoimmune diabetes. Clinical studies report that reduced programmed cell death protein 1 expression on helper T cells enhances cytotoxic T-cell activity, resulting in pancreatic β-cell destruction.^[5]^

ICI-DM is classified into ICI-associated type 1 diabetes or ICI-associated fulminant type 1 diabetes (IFD). The clinical features of ICI-DM align with loss of β-cell function, characterized by acute hyperglycemia, higher rates of ketosis, rapid decline in C-peptide levels, and increased glycemic variability.^[6,7]^ ICI-associated type 1 diabetes involves absolute insulin deficiency secondary to T-cell-mediated β-cell destruction and the presence of β-cell-specific serum autoantibodies.^[8,9]^ In contrast, IFD lacks detectable islet-specific autoantibodies^[10,11]^ and is more prevalent in East Asian populations. IFD presents with hyperglycemia, ketoacidosis, near-normal HbA1c, elevated pancreatic enzymes, severe insulin deficiency, and autoantibody negativity.^[12]^

Patients with ICI-DM are typically older than those with classic type 1 diabetes and often require intensive care management.^[13]^ The European Society for Medical Oncology recommends regular blood glucose monitoring for patients receiving ICIs.^[14]^ The American Society of Clinical Oncology advises baseline glucose testing at the start of each treatment cycle for 12 weeks, followed by monitoring every 3 to 6 weeks.^[15]^ The Japan Diabetes Society recommends glucose testing every 2 to 3 weeks during follow-up visits.^[16]^ Most ICI-DM patients require lifelong insulin therapy due to irreversible insulin deficiency.^[17,18]^ Although corticosteroids are used for other immune-related adverse events, they are ineffective for ICI-DM and may exacerbate hyperglycemia.^[18,19]^ Management of ICI-induced IFD follows type 1 diabetes guidelines,^[20]^ prioritizing basal-bolus insulin regimens to suppress gluconeogenesis and ketogenesis while covering prandial glucose excursions. For patients with malignant tumors or other conditions associated with limited life expectancy, or when the risks of treatment outweigh the benefits, the blood glucose management target may be appropriately adjusted to minimize the risk of hypoglycemia. Ideally, the HbA1c level should be maintained below 7.0%.^[20]^

Checkpoint inhibitors are molecules that modulate the signaling pathways responsible for immunological tolerance. The MAPK signaling pathway is essential for regulating proliferation, differentiation, apoptosis, and stress response. Relevant studies have confirmed that pMEK and pERK protein expressions were downregulated following programmed death ligand 1 knockdown.^[21]^ In IFD, excessive activation of MAPK-driven cytokine release can drive uncontrolled inflammation and organ damage. Downregulating the cascade reaction of this signaling pathway could theoretically reduce the speed of β-cell destruction. Targeting this biochemical pathway is fundamental for the development of therapeutic strategies for ICI-DM.^[22]^

Tislelizumab-induced IFD is exceptionally rare. Its pathogenesis overlaps with its antitumor mechanisms, making complete avoidance of this adverse effect clinically challenging. Moreover, ICI-associated diabetes with ketoacidosis often present with atypical symptoms that mimic tumor-related conditions, complicating early diagnosis. Therefore, regular glucose monitoring should be integrated into long-term follow-up protocols for patients receiving tislelizumab or similar ICIs. Enhancing patient adherence, educating on adverse drug reactions, and identifying predictive biomarkers for high-risk populations are critical goals for optimizing clinical practice in cancer immunotherapy.

## Acknowledgments

We sincerely express our gratitude to our patient and his daughter for their support and information.

## Author contributions

**Supervision:** Peng Jiang, Mingxiang Chen.

**Writing – original draft:** Xipeng Zhou.

**Writing –review & editing:** Jiashun Chen.
